# Conspiracy mentality in autistic and non-autistic individuals

**DOI:** 10.1080/13546805.2024.2399505

**Published:** 2024-09-10

**Authors:** Sanne Roels, Sander Begeer, Anke M. Scheeren, Jan-Willem van Prooijen

**Affiliations:** a Department of Clinical, Neuro and Developmental Psychology, Vrije Universiteit Amsterdam and Amsterdam Public Health Research Institute, Amsterdam, Netherlands; b Department of Experimental and Applied Psychology, Vrije Universiteit Amsterdam, Amsterdam, Netherlands

**Keywords:** Conspiracy theories, conspiracy mentality, autism

## Abstract

**Background:**

Belief in conspiracy theories has emerged across times and cultures. While previous accounts attributed conspiracy beliefs to mental health conditions, accumulating research suggests that conspiracy theories are common among the general population. In the present study we examined whether conspiracy mentality – that is, a general predisposition to believe conspiracy theories – differed between a group of autistic adults and a general population sample.

**Methods:**

This study included an autistic sample (*n *= 682) and a general population sample (*n* = 4358). Participants’ conspiracy mentality was measured using the Conspiracy Mentality Questionnaire (CMQ).

**Results:**

A one-way ANCOVA (controlling for participants’ age, gender, educational level, and ethnicity) revealed no difference in conspiracy mentality between an autism and a community sample.

**Conclusions:**

The current study suggests that being autistic, or having more autistic traits, does not predict conspiracy mentality. These findings underscore that autism does not predispose people to conspiracy theories and suggest that autism is neither a risk factor for, nor a protective factor against, conspiracy mentality.

## Introduction

Conspiracy theories are omnipresent in social media, websites, and public discourse. For instance, the COVID-19 pandemic gave rise to conspiracy theories suggesting that the corona virus is a human-made bioweapon (Imhoff & Lamberty, 2020) and a global strategy to enforce obligatory vaccination (Jensen et al., 2021). Although more visible than ever, belief in conspiracy theories is of all times, and present in all cultures investigated so far (Douglas et al., 2019; Hornsey, Bierwiaczonek, Sassenberg, & Douglas, 2023; van Prooijen & Douglas, 2018; van Prooijen & van Vugt, 2018; Van Prooijen, 2024). A conspiracy theory is the conviction that two or more actors (e.g. politicians) collude in secret agreement with the purpose of accomplishing some malicious goal (Douglas & Sutton, 2023). Conspiracy theories have a mostly negative influence on perceivers’ lives by stimulating poor health choices, deteriorating social relationships, and polarising societies (Van Prooijen, 2024). For example, conspiracy theories may instigate the belief that vaccines are dangerous, reducing believers’ willingness to vaccinate themselves or their children (Hornsey, Finlayson, Chatwood, & Begeny, 2020). Moreover, conspiracy theories reduce citizen’s civic virtue, such as their willingness to vote or reduce their carbon footprint (Jolley & Douglas, 2014). Given that conspiracy theories commonly exist in the general population (e.g. approximately half of the American people report to believe at least one conspiracy theory; Oliver & Wood, 2014), it is important to understand the psychological factors predicting belief in conspiracy theories.

Early conceptualisations assumed conspiracy beliefs to be rooted in mental health conditions (i.e. paranoia; Hofstadter, 1966). Indeed, in general population samples, conspiracy beliefs are associated with mental health (Darwin, Neave, & Holmes, 2011; Swami et al., 2011), including paranoia (Greenburgh & Raihani, 2022), narcissism (Cichocka, Marchlewska, Golec de Zavala, & Olechowski, 2016; Cichocka, Marchlewska, & Biddlestone, 2022), autistic traits (Georgiou, Delfabbro, & Balzan, 2021a; 2021b; 2022), and a wide range of personality disorders and psychopathological traits (for an overview, see Bowes, Costello, & Tasimi, 2023). These studies have contributed significantly to scientists’ understanding of the possible link of conspiracy beliefs with various mental health conditions. Yet, what is typically missing in research on a link between conspiracy beliefs and different mental health conditions is the inclusion of clinical samples. As conspiracy theories emerge frequently among people without psychopathology (Douglas et al., 2019; van Prooijen & Douglas, 2018), and conclusions based on general population samples cannot be automatically generalised to clinical samples, the lack of clinical samples precludes strong conclusions about the link between conspiracy beliefs and mental health conditions. The present study was designed to make a novel contribution to the literature by comparing a large group of autistic adults with a general population sample in their tendency to believe conspiracy theories.

One common finding in conspiracy theory research is that belief in one conspiracy theory strongly predicts belief in another, conceptually unrelated conspiracy theory (Williams, Marques, Hill, Kerr, & Ling, 2022; see also Abalakina-Paap, Stephan, Craig, & Gregory, 1999; Goertzel, 1994). These findings are integrated into the notion that, in a trait-like fashion, some people are more strongly inclined than others to attribute events in the world to the hostile actions of hidden conspiracies. This trait is referred to as conspiracy mentality (Bruder, Haffke, Neave, Nouripanah, & Imhoff, 2013; Imhoff & Bruder, 2014). While conspiracy mentality is typically heavily correlated with specific conspiracy beliefs (e.g. Covid-19 was created in the lab; 9/11 was an inside job), notable differences also exist between the two types of measures, making them suitable for different research purposes. As compared to specific conspiracy beliefs, conspiracy mentality is less driven by ideology and therefore better suited for comparison across different (e.g. cultural; ethnic; political) groups; it is more stable over time; and it has better psychometric properties (e.g. responses typically are more normally distributed). Specific conspiracy beliefs, in contrast, are more malleable, and thus more suitable for studying situational influences in experiments or longitudinal research (e.g. see Imhoff, Bertlich, & Frenken, 2022; Imhoff, 2024; Nera, 2024; Sutton, Douglas, & Trella, 2024). Given the present focus on whether autistic versus non-autistic people structurally differ in their general tendency to endorse conspiracy beliefs, we focused on conspiracy mentality in the current project.

We evaluate two opposing ideas: An autism diagnosis is a *risk* for increased conspiracy mentality (i.e. autism as risk factor), versus autism *protects* against conspiratorial mentality (i.e. autism as protective factor). Below, we elaborate on both ideas in more detail.

### Autism as a risk factor for conspiracy mentality

One common insight is that aversive social experiences increase people’s susceptibility to conspiracy theories. For instance, feelings of anxiety and marginalisation prompt an epistemic sense-making process that facilitates conspiracy beliefs (Douglas et al., 2019; van Prooijen & Douglas, 2018). Likewise, feelings of social exclusion and relative deprivation may lead people to blame their lack of opportunities on a “rigged” system. As shown by Poon and colleagues (2020), excluding people from social interactions also increases conspiracy beliefs. Resulting from feelings of relative deprivation, marginalised minority members (e.g. ethnic minorities) are more susceptible to conspiracy theories (van Prooijen, Staman, & Krouwel, 2018).

Such aversive social experiences are common among people with autism (Han, Scior, Avramides, & Crane, 2022). Autistic individuals may experience stigmatisation often, which can lead to feelings of social isolation and anxiety (Cage, Di Monaco, & Newell, 2018; Han et al., 2022). This stigma excludes some autistic people from full participation in society. Indeed, even in high income countries autistic individuals are more often unemployed compared to other disability groups, such as those with an intellectual disability (Bury, Hedley, Uljarević, Stokes, & Begeer, 2024; Shattuck et al., 2012). Thus, being autistic makes people prone to negative social experiences such as social exclusion, a limited social support network, reduced career opportunities, and discrimination. Together these findings suggest that the social problems, stigmatisation, and societal exclusion commonly experienced by autistic individuals may stimulate a high conspiracy mentality. Following this same line of reasoning, autistic individuals with relatively many autism traits tend to experience a higher degree of stigma than those expressing fewer autism traits (Turnock, Langley, & Jones, 2022), resulting in higher conspiracy mentality among individuals with many autism traits. Indeed, studies in regular population samples indicate a positive relationship between autistic traits and conspiracy beliefs (Georgiou et al., 2021a; 2021b; 2022).

### Autism as protective factor against conspiracy mentality

An alternative line of reasoning suggests that autism might in fact be a protective factor. While earlier research found a positive link between autistic traits and conspiracy beliefs despite them being positively associated with some of these protective factors (e.g. analytic thinking; systematic information search), this study failed to include people with a clinical diagnosis of autism (Georgiou, Delfabbro, & Balzan, 2021b). A core cognitive feature of autistic people is that they display an analytic thinking style. For instance, autistic individuals often show reduced intuitive thinking and more analytic reasoning compared to non-autistic people (Birmingham, Stanley, Nair, & Adolphs, 2015; Brosnan, Ashwin, & Lewton, 2017; De Martino, Harrison, Knafo, Bird, & Dolan, 2008). However, an analytic cognitive style does not directly translate into a non-conspiracy attitude. Other factors, including the type and credibility of sources of information, play a significant role here as well (Tarasi, Borgomaneri, & Romei, 2023).

In a comparison of the opposing ideas that conspiracy beliefs are a form of rational scepticism versus a form of gullibility, research overwhelmingly supports the gullibility hypothesis (Van Prooijen, 2019). This implies that the cognitive features commonly associated with autism predicts reduced conspiracy mentality. Indeed, analytic thinking is associated with reduced conspiracy beliefs, and instead, conspiracy beliefs are associated with a more intuitive thinking style (Swami, Voracek, Stieger, Tran, & Furnham, 2014). Moreover, stronger conspiracy beliefs are related to the tendency to make quick decisions based on limited evidence, known as the jumping-to-conclusions bias (Denovan, Dagnall, Drinkwater, Parker, & Neave, 2020; Pytlik, Soll, & Mehl, 2020; Swami et al., 2014). Autistic individuals generally take a more rational, contemplative approach (Brosnan et al., 2017), and as such, they may have an analytic thinking style that naturally predisposes them to scepticism about conspiracy theories. Thus, being autistic might protect against a high conspiracy mentality.

### The current research

The current research aims to evaluate two opposing hypotheses stating that autistic individuals either have a higher (“autism as risk factor”) or lower conspiracy mentality (“autism as protective factor”) in comparison to non-autistic people. We assessed these hypotheses in a preregistered study by comparing a large sample of autistic people with a large random population sample.

## Method

### Participants

The current study included 5040 participants, of whom 682 were autistic and 4358 belonged to a Dutch community sample (see Table 1 for sample characteristics for the autism and community sample separately). All autistic participants reported a clinical diagnosis of ASD (according to DSM-criteria) which was ascertained by a team of qualified clinicians, independent of the study (Scheeren, Buil, Howlin, Bartels, & Begeer, 2022). The age at which the autistic participants received their ASD diagnosis ranged from 2 to 75 years old (*M *= 37.24, *SD* = 15.26). The autistic participants were younger (*M *= 46.74, *SD *= 13.77) than the participants from the community sample (*M* = 54.22, *SD* = 16.14), *t* (5083) = −11.48, *p* < .001) and the autism sample included relatively more women (55.3%) compared to the community sample (38.8%) (Χ^2^ (2, N = 5040) = 100.70, *p *< .001). Autistic participants less often reported a migration background (8.1%) than participants in the community sample (15,1%) (χ^2^ (1, *N* = 4821) = 23.92, *p *< .001) and relatively more autistic participants were highly educated compared to controls (Χ^2^ (2, *N* = 4655) = 25.18, *p *< .001). These variables (age, gender, ethnicity, and educational level) are all included as covariates in the primary analyses.

**Table 1.** Demographic characteristics of participants.

	Autistic sample (*n *= 682)	Community sample (*n* = 4358)
Age (*M*, *SD*)	46.74 (13.77)	54.22 (16.14)
Gender (*n*, %)		
Man	299 (43.8%)	2668 (61.2%)
Woman	377 (55.3%)	1689 (38.8%)
Other	6 (0.9%)	1 (0%)
Educational Level ^a^		
Low	69 (13.4%)	683 (16.5%)
Middle	170 (33%)	1719 (41.5%)
High	276 (53.6%)	1738 (42%)
Ethnicity		
Dutch	626 (91.9%)	3514 (84.9%)
Non-Dutch ^b^	55 (8.1%)	626 (15.1%)
Autistic traits AQ-28 total score	84.41 (10.87)	N/A

*M*: mean; *SD*: standard deviation; *N.A.*: not available

^a^ 167 participants (24.5%) of the autistic sample and 218 participants (5%) of the community sample did not provide data on their educational level.

^b^ Participants who reported to have at least one parent who was not born in the Netherlands.

An a priori sensitivity analysis using G*Power/version 3.1.9.6 showed that with the sample size even an effect size (*f)* as small as 0.05 could be detected with an alpha level of 0.05 and a power of 0.95 (Faul, Erdfelder, Lang, & Buchner, 2007).

### Procedure

Data of the autistic sample were obtained from the Netherlands Autism Register (NAR). The NAR is a research initiative from the Dutch Association for Autism (Nederlandse Vereniging voor Autisme; NVA) and the Vrije Universiteit Amsterdam. It provides a database of individuals with an autism diagnosis who are invited to fill out online questionnaires every year. The conspiracy mentality survey was part of a larger study focused on the effects of the Covid-19 pandemic on autistic adults (see Scheeren, Howlin, Pellicano, Magiati, & Begeer, 2022). All data from the NAR are either self-reported or parent-reported (in case the autistic individual is younger than 16 years). Upon registration, participants sign an online informed consent form. The Medical Ethical Committee of the XXX (blinded) approved of the study. More detailed information on the NAR: https://www.nederlandsautismeregister.nl/english/

The community sample data were obtained through an online survey on a panel by Kieskompas (“Election Compass”), a political research agency in the Netherlands. Kieskompas coordinates large research panels for which people register voluntarily through online Voting Advice Applications (VAAs). Kieskompas adheres to GDPR (i.e. EU privacy) regulations, is closely monitored by the Dutch privacy authority, and acts in line with the ethical norms of VU Amsterdam. More information can be found at https://www.kieskompas.nl/en/about/ The survey was fielded on a panel pre-stratified to be representative for the Dutch population on the benchmarks gender, age, education, ethnicity, region, and voting behaviour. Preregistration of this study can be found at Open Science Framework (10.17605/OSF.IO/BHE28).

### Measures

## Demographics

Demographic variables including participants’ gender (man, woman, other), age, educational level, and ethnicity (i.e. parents’ country of birth) were assessed.

## Conspiracy mentality

Conspiracy mentality was measured using the Conspiracy Mentality Questionnaire (CMQ; Bruder et al., 2013). The CMQ is a 5-item self-report questionnaire assessing participant’s general susceptibility to believe in conspiracy theories. Items include statements such as “I think that many very important things happen in the world, which the public is never informed about”. Participants can rate their agreement with the statement on an 11-point Likert scale, ranging from 1 (“definitely not”) to 11 (“definitely yes”). Total scores are computed by averaging the sum of all item scores, with higher scores reflecting a stronger conspiracy mentality.

## Autistic traits

The extent to which an individual shows traits associated with the autism spectrum was measured with a 28-item version of the Autism Spectrum Quotient (AQ; Hoekstra et al., 2011). Each item consists of a statement (e.g. “I find social situations easy”) on which the participants can index their agreement on a 4-point Likert-scale (ranging from “definitely agree”, “slightly agree”, “slightly disagree” to “definitely disagree”). Subscale scores can be computed by adding all item scores belonging to that subscale; the total score is the sum of all subscale scores. Total AQ-28 scores range from 28 to 112, with higher total scores indicating the presence of more autistic traits. The AQ-28 has previously shown acceptable reliability (α = between 0.77 and 0.79; Hoekstra et al., 2011) and demonstrated excellent reliability in the current study (α = 0.93).

### Data analyses

To determine whether autistic individuals and the community sample differed significantly in conspiracy mentality, a one-way ANCOVA was conducted. Considering that educational level and ethnicity are associated with a higher conspiracy mentality (van Prooijen, 2017; van Prooijen et al., 2018) and taking significant group differences in participants’ age, gender, educational level, and ethnicity into account (see Table 1), all these variables were added as covariates in the analysis.

## Results

### Main analysis

**Figure 1.**
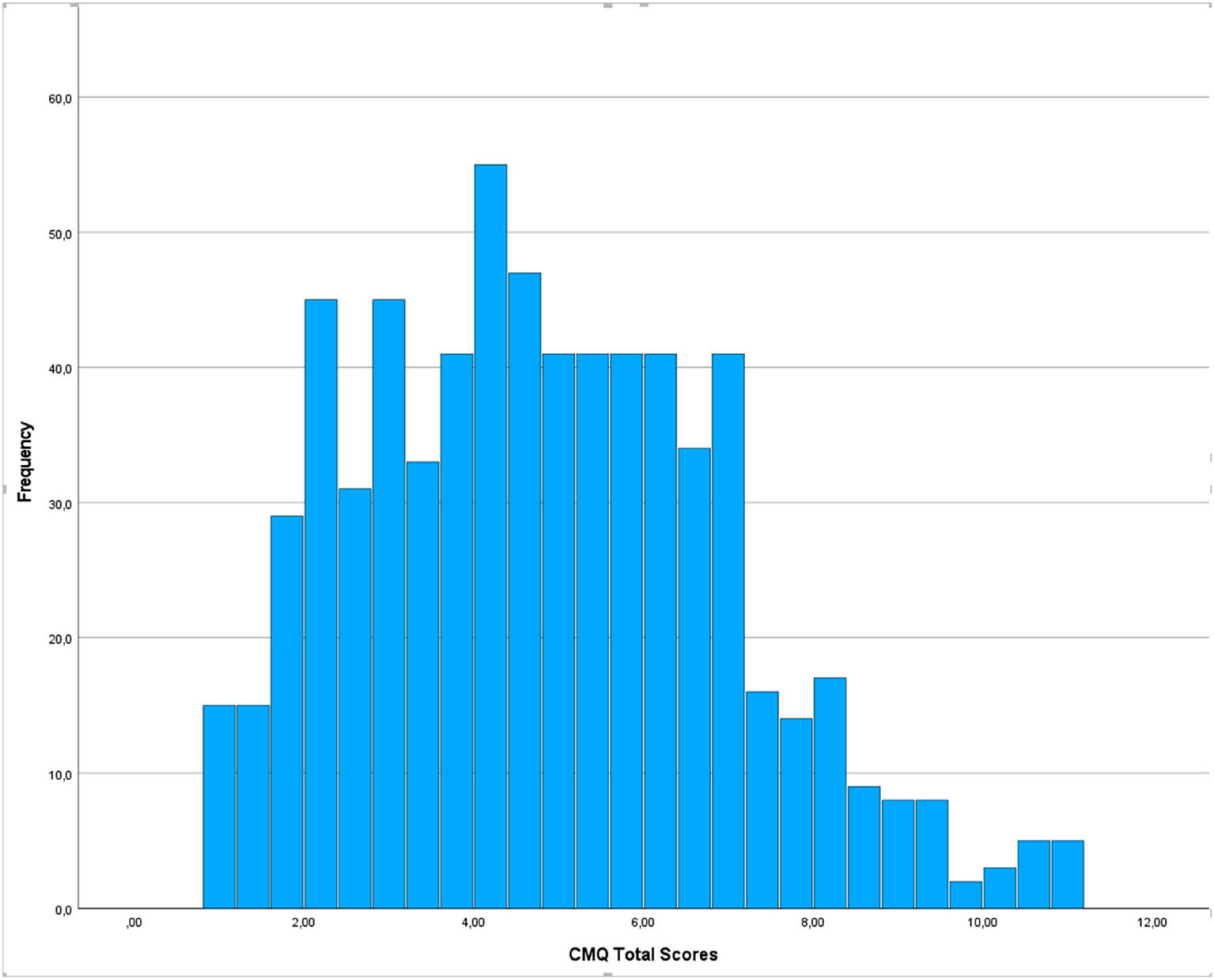
Frequencies CMQ total scores in autistic sample.

**Figure 2.**
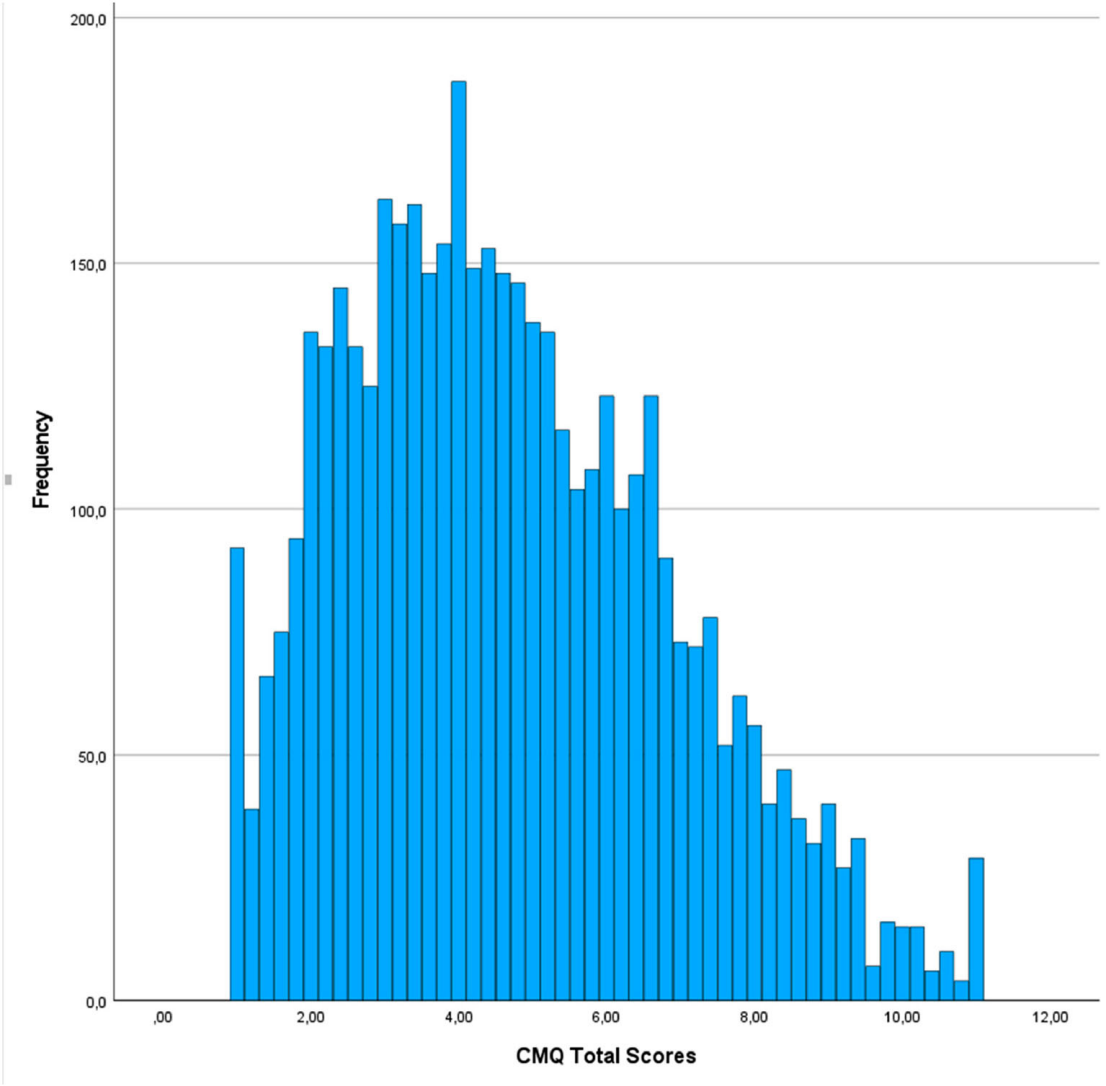
Frequencies CMQ total scores in community sample.

After ascertaining the normality of the data by visually expecting the Q-Q plots in the autistic sample and the community sample separately (supplementary material: Figures 1 and 2, respectively), Bartlett’s test demonstrated that the variances of CMQ total scores were equal across groups (B(1) = 0.280, *p* = .0.596. < χ^2^ (3.84)). Counter to our two opposing hypotheses, the one-way ANCOVA showed similar levels of conspiracy mentality in autistic adults and the community sample (*F* (1, 4648) = 0.081, *p* = 0.776), indicating no difference in conspiracy mentality between the autistic (*M* = 4.66, *SD* = 2.10) and the community sample (*M* = 4.70, *SD* = 2.20; Figures 1 and 2, respectively). All covariates were significant: participants’ younger age, male gender, lower education, and non-Dutch ethnicity were associated with higher CMQ scores (Table 2).

**Table 2.** Covariates of the one-way ANCOVA.

Covariate	*F*	*Sig*	*Partial η^2^*	*Direction of effect*
Age	9.30	0.002	0.002 ^a^	younger > older
Gender	43.30	0.002	0.002	men > women
Education	73.33	0.000	0.016	low > middle > high
Ethnicity	36.14	0.006	0.002	non-Dutch ^b^ > Dutch

*Sig*: significance.

^a^ small, medium, and large effects would be reflected in partial *η*^2^ values of 0.01, 0.06 and 0.14, respectively (Richardson, 2011).

^b^ Participants who reported to have at least one parent who was not born in the Netherlands.

### Exploratory analyses

An additional equivalence test was performed to check for the robustness of the non-significant group difference in conspiracy mentality as shown by the ANCOVA. We examined whether the group difference fell between prespecified boundaries of a smallest effect size of interest (Lakens, Scheel, & Isager, 2018). We constructed a 90% confidence interval (CI) for our effect (Cohen’s *d*) and set the upper boundary at 0.2 and the lower boundary at – 0.2, based on Cohen’s criteria of a small effect size. The computed 90% CI [−0.084–0.051] did not exceed this lower and upper boundary, therefore demonstrating equivalence in conspiracy mentality score between groups, and providing strong support for the null hypothesis.

Within the autistic sample, we conducted two exploratory regression analyses to examine whether the degree of autistic traits and the age of autism diagnosis predicted conspiracy mentality. Autistic traits, i.e. AQ-28 total scores, *β* = .08, *p* = 0.06, and age of autism diagnosis, *β* = 0.02, *p *= 0.62, did not predict CMQ total scores, *R*^2^ = .007, *F*(2, 612) = 2.23, *p* = .11. However, a regression model including all five AQ-28 subscales indicated that the scales ‘*Numbers and Patterns’*, *β* = .11, *p* < 0.01, and ‘*Imagination’, β* = -.09, *p* < 0.05 predicted CMQ scores, *R*^2^ = .03, *F*(6, 608) = 3.14, *p* < .01. Autistic participants with a higher Number and Patterns score tended to report a stronger conspiracy mentality, while those with higher scores on Imagination were less likely to endorse a conspiracy mentality.

## Discussion

Expanding on previous work (Georgiou et al., 2021; 2022), the present study compared conspiracy mentality of autistic adults with that of a community sample. We contrasted two ideas: (a) the autistic group would either show a stronger conspiracy mentality (i.e. autism as *risk* factor) or (b) a weaker conspiracy mentality (i.e. autism as *protective* factor) than the community sample. Using a well-powered design, and controlling for a range of potential confounders, both groups showed a strikingly similar level of conspiracy mentality. Additional equivalence testing as well as exploratory regression analyses within the autistic sample, further supported the null hypothesis. These findings suggest that there is no link between autism and conspiracy mentality.

While autistic traits in general did not predict conspiracy mentality in the autism sample, the specific AQ-28 subscales Numbers and Patterns and Imagination did. This suggests that the inclinication to focus on details and patterns, rather than the full spectrum of autistic traits, could be associated with a conspiracy mentality. Possibly, a strong tendency to focus on specific patterns may cause some people to ‘get lost’ in the details and loose the general overview of information. Conversely, a conspiracy mentality may be associated with seeing specific associations and patterns. Imagination, as operationalised by the AQ, includes the ability to work out other peoples’ intentions and imagine what it is like to be someone else. In some cases high imagination and empathy levels may lead to overinterpretation and paranoia, as can be seen in people with psychosis spectrum disorders, which might explain the link with conspiracy mentality. However, these associations were small and based on cross-sectional correlations, and should therefore first be replicated to justify futher interpretation.

The present findings contribute to the ongoing debate on the potentially pathological qualities of conspiracy beliefs. Early writings on the topic have assumed that a certain level of pathology is necessary for people to believe in conspiracy theories (Hofstadter, 1966). However, more recent work suggests that conspiracy beliefs are common among the general population, and that people who have no mental health condition may also believe conspiracy theories (Oliver & Wood, 2014). Our findings contribute to this debate by suggesting that being autistic does not put people at risk for, nor protects against, a general tendency to perceive conspiracies in the world. Of course, this does not preclude the possibility that other forms of pathology are associated with conspiracy beliefs. Indeed, various subclinical traits are reliably associated with conspiracy beliefs, suggesting a need to further investigate these issues in clinical samples (e.g. Bowes et al., 2023; Cichocka et al., 2022; Greenburgh & Raihani, 2022).

The ‘autism as risk hypothesis’ was based on the reported higher rates of anxiety, stigmatisation, and societal exclusion of autistic people compared to non-autistic people, which have all been linked to increased conspiracy mentality (e.g. Douglas et al., 2019; Poon, Chen, & Wong, 2020). Although our findings do not support a link between autism and conspiracy mentality, further in-depth research is needed to examine the potential link between autistic individuals’ experience of societal exclusion and stigmatisation on the one hand and conspiracy mentality on the other. The ‘autism as protection hypothesis’ assumed that the rational, analytic qualities of autistic individuals may make them less vulnerable to conspiracy mentality. The current lack of evidence suggests that despite autistic people’s tendencies towards analytic thinking, autism does not protect them from conspiracy beliefs (cf. Georgiou, Delfabbro, & Balzan, 2022). These cognitive qualities may therefore be unrelated to conspiracy mentality among people with autism. Alternatively, while no evidence was found for either the risk or protection hypothesis, it is possible that both mechanisms may have had a dampening effect on each other, thus explaining our null finding.

The current study has several strengths and limitations. Among the strengths are the preregistration of the study, in combination with a large autistic group and a large community sample. Moreover, standardised, and validated measures were used to assess conspiracy mentality (Bruder et al., 2013) and autistic traits (Hoekstra et al., 2011). As such, this study offers a strong test of a relationship between autism and conspiracy mentality, yet we did not detect any evidence for this link.

A limitation of the present study is that our assumptions about group differences in cognitive preference, stigmatisation, social exclusion, and analytical thinking were not directly assessed in our samples. Furthermore, it remains possible that specific mental health conditions make people more vulnerable to conspiracy mentality. Additionally, we only focused on general conspiracy mentality, and therefore cannot exclude the possibility that autistic people differ from non-autistic people in more specific conspiracy beliefs (e.g. beliefs that the corona virus is a hoax). Indeed, while conspiracy mentality and conspiracy beliefs are strongly correlated, important conceptual and psychometric differences exist between these diverse types of measurement (Imhoff et al., 2022; Imhoff, 2024; Nera, 2024; Sutton et al., 2024). Future research should address these issues by comparing other clinical populations to a community sample, and by including other measures of belief in conspiracy theories. Moreover, future research may more elaborately examine the cognitive and affective variables that moderate a potential link between psychopathology and conspiracy beliefs.

In addition, we lacked information regarding clinical diagnoses, mental health problems, and autistic traits in our general population sample. Based on an autism prevalence rate of 1-2% (Zeidan et al., 2022), it is likely that the comparison group also contained some, though not many autistic individuals. Furthermore, the autistic participants in our study were relatively highly educated, were more often female, and did not have a migration background as often as compared to citizens in the Netherlands. Yet, we covaried for these factors. Furthermore, many of our autistic participants received their autism diagnosis in adulthood. One might therefore argue that they may not have faced as many aversive social experiences and stigma as people who received their diagnosis at a younger age. This latter concern is mitigated, however, because age of autism diagnosis did not correlate with conspiracy mentality score.

Concluding, the current research provides no evidence that believing in conspiracy theories is linked to autism or self-reported autistic traits, as we observed no differences in conspiracy mentality between autistic individuals and a general population sample, nor did we find an association between autistic traits in general and conspiracy mentality among autistic individuals. Autism is neither a risk factor nor a protective factor against a conspiracy mentality.

## Authorship contribution

**Sanne Roels:** Writing – Original draft preparation, Methodology, Analyses. **Sander Begeer**: Conceptualisation, Writing – Reviewing and Editing, Supervision, Methodology, Analyses **Anke Scheeren**: Conceptualisation, Writing – Reviewing and Editing, Methodology, Analyses. **Jan-Willem van Prooijen**: Conceptualisation, Writing – Reviewing and Editing, Supervision, Methodology, Analyses.

## Ethical standards

The authors assert that all procedures contributing to this work comply with the ethical standards of the relevant national and institutional committees on human experimentation and with the Helsinki Declaration of 1975, as revised in 2008.

## Acknowledgment

The authors thank all participants from the Netherlands Autism Register (NAR) and Kieskompas.

## Data Availability

Data available on request from the authors. Due to the privacy-sensitive qualities of the autistic sample, it is impossible for us to share the data through a public repository. However, it is possible to receive a copy of the data upon request from the authors on the condition of signing a non-disclosure agreement.
